# Influence of dietary iodine deficiency on the thyroid gland in *Slc26a4*-null mutant mice

**DOI:** 10.1186/1756-6614-4-10

**Published:** 2011-06-20

**Authors:** Tomoyuki Iwata, Tadao Yoshida, Masaaki Teranishi, Yoshiharu Murata, Yoshitaka Hayashi, Yasuhiko Kanou, Andrew J Griffith, Tsutomu Nakashima

**Affiliations:** 1 Department of Otorhinolaryngology Nagoya University Graduate School of Medicine, 65 Tsurumai-cho, Showa-ku, Nagoya, Aichi 466-8550, Japan; 2 Inazawa City Hospital, 1-1 Gokusho-cho, Inazawa, Aichi 492-8510, Japan; 3 Department of genetics, Research Institute of Environmental Medicine, Nagoya University, Furo-cho, Chikusa-ku, Nagoya, Aichi 464-8601, Japan; 4 National Institute on Deafness and Other Communications Disorders, National Institutes of Health, 5 Research Court, Rockville, MD 20850 USA

## Abstract

**Background:**

Pendred syndrome (PDS) is an autosomal recessive disorder characterized by sensorineural hearing impairment and variable degree of goitrous enlargement of the thyroid gland with a partial defect in iodine organification. The thyroid function phenotype can range from normal function to overt hypothyroidism. It is caused by loss-of-function mutations in the *SLC26A4 *(*PDS*) gene. The severity of the goiter has been postulated to depend on the amount of dietary iodine intake. However, direct evidence has not been shown to support this hypothesis. Because *Slc26a4*-null mice have deafness but do not develop goiter, we fed the mutant mice a control diet or an iodine-deficient diet to evaluate whether iodine deficiency is a causative environmental factor for goiter development in PDS.

**Methods:**

We evaluated the thyroid volume in histological sections with the use of three-dimensional reconstitution software, we measured serum levels of total tri-iodothyronine (TT3) and total thyroxine (TT4) levels, and we studied the thyroid gland morphology by transmission electron microscopy.

**Results:**

TT4 levels became low but TT3 levels did not change significantly after eight weeks of an iodine-deficient diet compared to levels in the control diet animals. Even in *Slc26a4*-null mice fed an iodine-deficient diet, the volume of the thyroid gland did not increase although the size of each epithelial cell increased with a concomitant decrease of thyroid colloidal area.

**Conclusions:**

An iodine-deficient diet did not induce goiter in *Slc26a4*-null mice, suggesting that other environmental, epigenetic or genetic factors are involved in goiter development in PDS.

## Background

Pendred syndrome (PDS) is an autosomal recessive disorder characterized by sensorineural hearing impairment, presence of goiter, and a partial defect in iodine organification [1]. The goiter in PDS is variable in its presentation; it can develop at any age (although generally after puberty), but may be totally absent in some affected individuals [2]. Also, there is substantial intrafamilial and regional variation, and nutritional iodine intake may be a significant modifier of the thyroid phenotype [1]. Kopp *et al*. suggested that under conditions of sufficient iodine intake, thyroid enlargement may be very mild or absent, and hence these patients are often simply categorized as having enlarged vestibular aqueduct [1]. Sato *et al*. also suggested that even in patients with impaired iodide transport, high iodine intake may prevent the development of goiter [3].

*Slc26a4*-null (*Slc26a4^-/-^*) mutant mice were generated by Everett *et al*. [2]. *Slc26a4^-/- ^*mice are profoundly deaf with vestibular dysfunction, but they lack goiter and thyroid histological abnormalities. We hypothesized that the absence of goiter and hypothyroidism in *Slc26a4^-/- ^*mice was due to a sufficient iodine intake, and that goiter and hypothyroidism might be induced by iodine deficiency. We, therefore, performed this study to investigate the influence of iodine intake on serum thyroid hormone levels and the histology and volume of the thyroid gland in *Slc26a4^-/- ^*mice.

## Materials and methods

### *Slc26a4*-null mice

An *Slc26a4*-null (*Slc26a4^-/-^*) mouse colony was established and bred with homozygotes and heterozygotes imported from the National Institutes of Health (Rockville, Maryland) [2]. The line was maintained on a 129/SvEv background.

### Breeding and Diet

Matings were performed between *Slc26a4^-/- ^*and *Slc26a4^-/-^*, and between *Slc26a4^+/- ^*and *Slc26a4^+/- ^*mice. These mice were fed a control diet (CLEA Japan Inc.). F1 offspring at two months of age were paired for mating. The mice were fed iodine-deficient chow (CLEA Japan Inc. T-08514) or control chow (CLEA Japan Inc. T-08513) from the beginning of the mating. Each chow was comprised solely of artificial materials. According to an analysis by the Laboratories for Food & Environmental Science, Tokyo, Japan, the iodine level was less than the sensing threshold (< 0.02 mg%) in iodine-deficient chow (ICD) whereas it was 0.51 mg% in control chow (CCD). F2 offspring were fed with the same diet as their parents for 12 to 16 weeks after weaning. *Slc26a4 *genotyping was performed on DNA prepared from tail specimens obtained at the time of sacrifice of the mice. There were six groups comprising one of three different genotypes (*Slc26a4^-/-^*, *Slc26a4^+/-^*, and *Slc26a4^+/^*) and either of ICD or CCD. Thirty-one 12 to 16 week-old males were used for this study (Table 1). Females were not analyzed in order to avoid the effect of menstrual cycles on hormone levels. The experimental protocol was approved by the Experimental Animal Management Committee, Nagoya University, Graduate School of Medicine.

**Table 1:** Total tri-iodothyronine (TT3) and total thyroxine (TT4) levels and thyroid volumes in *Slc26a4*-null mice eating control chow (CCD) or iodine-deficient chow (ICD).

Genotype	Diet	Body weight (g)	TT3 (ng/ml)	TT4 (μg/dl)	Volume (mm^3^)
*Slc26a4^-/-^*	CCD	25.6	2.80	8.70	0.95
		
		22.5	1.24	5.10	1.03
		
		19.8	0.74	4.68	EM
		
		28.9	0.76	3.99	2.27
		
		29.7	0.78	3.78	2.93
	
	ICD	25.4	0.87	3.60	0.67
		
		22.5	0.79	3.58	1.28
		
		21.3	0.99	2.77	0.88
		
		23.0	1.01	3.52	1.28
		
		20.9	0.86	3.42	0.75
		
		25.7	1.04	3.12	1.07
		
		24.6	0.88	1.79	EM

*Slc26a4^+/-^*	CCD	25.6	1.30	6.30	1.48
		
		23.8	1.35	5.90	1.47
		
		23.5	1.45	4.03	1.28
		
		27.4	2.40	8.10	1.03
		
		28.2	1.60	5.05	2.82
		
		25.1	0.82	3.83	EM
		
		38.0	0.81	4.13	3.22
	
	ICD	24.8	1.02	4.29	1.31
		
		23.4	0.84	3.11	0.66
		
		29.2	0.87	2.64	EM
		
		33.3	0.82	2.41	2.04
		
		31.7	1.12	2.92	1.92

*Slc26a4^+/+^*	CCD	26.8	1.55	3.97	1.63
		
		24.5	1.60	3.88	1.44
		
		23.7	2.10	8.40	1.38
		
		25.0	1.60	5.95	1.31
		
		26.7	0.79	3.45	1.24
	
	ICD	23.4	0.93	4.26	1.11
		
		23.8	1.20	4.35	UM

EM, examined with electron microscopy.

UM, unmeasurable due to failure to prepare adequate thin sections.

### Serum thyroid hormones

After deep anesthesia by intraperitoneal injection of pentobarbital sodium, blood was collected from the inferior vena cava. Serum total tri-iodothyronine (TT3) and total thyroxine (TT4) levels were measured by an electrochemiluminescence immunoassay (TT3: DRG^® ^T-3 ELISA, TT4: DRG^® ^T-4 ELISA, DRG International, East Mountainside, New Jersey USA).

### Thyroid histology and volume

After intracardiac infusion of 4% paraformaldehyde, thyroid glands were excised together with adjacent tracheae and immersed in the same fixative for 24 hours at 4°C. The specimens were placed in 10% EDTA for seven days, washed with phosphate buffered saline (PBS), embedded in paraffin, and sectioned at 4-μm thickness for collection of every fifth section. The sections were stained with hematoxylin-eosin. The serial sections were observed with a light microscope system (BZ-8000, Keyence, Tokyo, Japan) and saved as digital images. A digital image of the whole thyroid was reconstructed and the volume was measured using three-dimensional reconstruction software, ZedView (LEXI, Tokyo, Japan).

### Ultrastructural evaluation

One mouse was selected randomly for electron microscopic observation of the thyroid gland from each group of *Slc26a4^-/- ^*CCD, *Slc26a4^-/- ^*ICD, *Slc26a4^+/-^*CCD and *Slc26a4^+/- ^*ICD. The electron microscopic observation was done according to the method described previously [4]. Two-mm^3 ^thyroid specimens were excised, fixed in 2.5% glutaraldehyde for 24 hours at 4°C, washed in 0.1M phosphate buffer (pH = 7.0), and fixed again in 1% osmium tetroxide for 3 hours at 4°C. The samples were dehydrated in a graded series of ethanol and embedded in epoxy resin. Ultrathin sections were cut, double stained with uranyl acetate and lead citrate, and examined using a JEOL JEM100S electron microscope (JEOL, Tokyo, Japan).

### Statistics

Statistical analysis was performed using SPSS Statistics ver.19.0 (SPSS Inc., Chicago, IL). One-way ANOVA and Mann-Whitney U-testing were used for statistical analysis. A *P *value less than 0.05 defined a significant difference.

## Results

### Volume of thyroid gland

**Figure 1:**
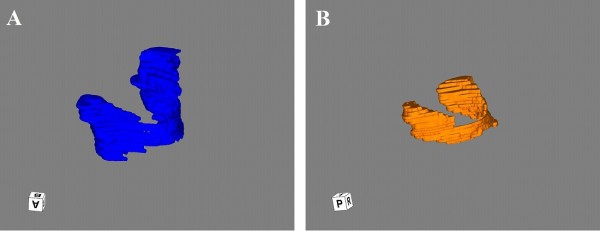
**The thyroid images reconstructed by three-dimensional reconstruction software**. A, *Slc26a4^-/- ^*mouse thyroid with iodine-deficient chow (ICD) (reconstructed with three-dimensional software, Zed View). B, *Slc26a4^-/- ^*mouse thyroid with control chow (CCD) (reconstructed with three-dimensional software, Zed View).

The thyroid volume in each animal is shown in Table 1. Mean thyroid volumes of *Slc26a4^-/-^*, *Slc26a4^+/- ^*and *Slc26a4^+/+ ^*mice fed with CCD were 1.8 ± 1.0 mm^3^, 1.9 ± 0.9 mm^3^, 1.4 ± 0.2 mm^3 ^respectively. The mean thyroid volumes of *Slc26a4^-/-^*, *Slc26a4^+/- ^*and *Slc26a4^+/+ ^*mice fed with ICD were 1.0 ± 0.3 mm^3^, 1.5 ± 0.6 mm^3^, 1.1 mm^3 ^respectively. There were no significant differences in mean thyroid volumes between ICD and CCD groups for any genotype. The thyroid images reconstructed by three-dimensional reconstruction software are shown in Figure 1.

### Histological findings

**Figure 2:**
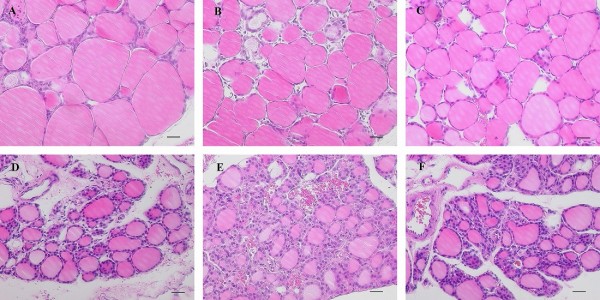
**Light microscopic findings of the thyroid gland**. A, *Slc26a4^-/- ^*control chow (CCD); B, *Slc26a4^+/- ^*CCD; C, *Slc26a4^+/+ ^*CCD; D, *Slc26a4^-/- ^*iodine-deficient chow (ICD); E, *Slc26a4^+/- ^*ICD; F, *Slc26a4^+/+ ^*ICD. Scale bars: 30 μm.

**Figure 3:**
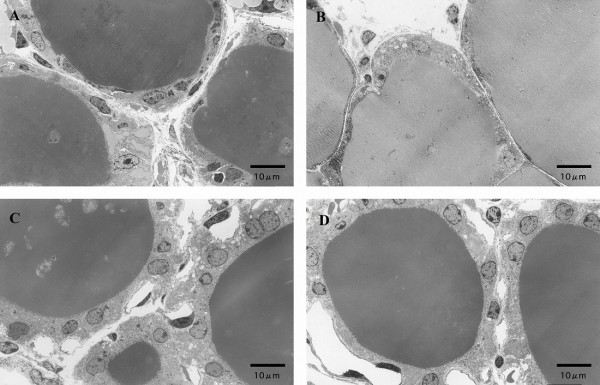
**Electron microscopic findings of the throid gland**. A, *Slc26a4^-/- ^*CCD; B, *Slc26a4^+/- ^*CCD; C, *Slc26a4^-/- ^*ICD; D, *Slc26a4^+/- ^*ICD.

Figure 2 demonstrates light microscopic observation of the thyroid gland of ICD and CCD groups for the three different *Slc26a4 *genotypes. The size and height of epithelial cells increased with a concomitant decrease of colloidal area in ICD thyroid glands as compared to those of CCD animals among all genotypes. Electron microscopic observations in *Slc26a4^-/- ^*and *Slc26a4^+/- ^*thyroid glands were consistent with these findings (Figure 3).

### Serum thyroid hormone levels

Serum concentrations of TT3 and TT4 in each animal are shown in Table 1. In the CCD group, the average TT3 levels were 1.26 μg/dl, 1.39 μg/dl and 1.53 μg/dl in *Slc26a4^-/-^*, *Slc26a4^+/- ^*and *Slc26a4^+/+ ^*mice, respectively. In the ICD group, the average TT3 levels were 0.92 μg/dl, 0.93 μg/dl and 1.07 μg/dl in *Slc26a4^-/-^*, *Slc26a4^+/- ^*and *Slc26a4^+/+ ^*mice, respectively. The average TT4 levels in the CCD group were 5.25 μg/dl, 5.33 μg/dl and 5.13 μg/dl in *Slc26a4^-/-^*, *Slc26a4^+/- ^*and *Slc26a4^+/+ ^*mice, respectively. The average TT4 levels in the ICD group were 3.11 μg/dl, 3.07 μg/dl and 4.31 μg/dl in *Slc26a4^-/-^*, *Slc26a4^+/- ^*and *Slc26a4^+/+ ^*mice, respectively. One-way ANOVA did not reveal a significant difference in TT3 and TT4 levels among the three genotypes.

**Figure 4:**
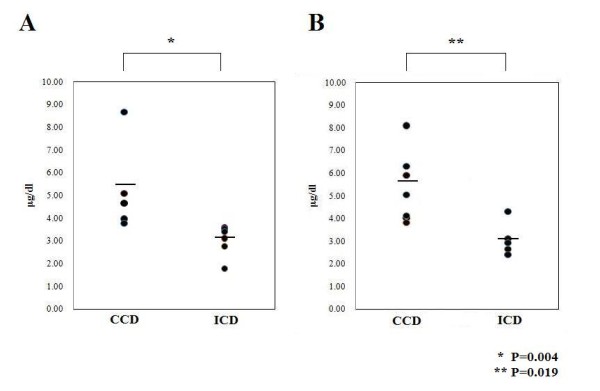
**Effect of iodine deficiency on serum total thyroxine (TT4) levels in Slc26a4^-/-^ and Slc26a4^+/-^ mice.**  A, TT4 levels of Slc26a4^-/-^ mice among control chow (CCD) and iodine-deficient chow (ICD) groups.   B, TT4 levels of Slc26a4^+/-^ mice among CCD and ICD groups.   Mann-Whitney U-testing indicated significant differences for both comparisons.

As shown in Figure 4, Mann-Whitney U-testing revealed that serum TT4 level was lower in the ICD group than in the CCD group both in *Slc26a4^-/- ^*and *Slc26a4^+/- ^*mice (*p *= 0.004 and *p *= 0.019, respectively). In *Slc26a4^+/+ ^*mice, Mann-Whitney U-testing was not adequate to compare between ICD group and CCD group because the number of ICD animals was two. On the other hand, the TT3 level was not different significantly between the ICD and CCD groups.

## Discussion

Mutations of the *SLC26A4 *(*PDS*) gene can cause sensorineural hearing loss with goiter (PDS) or non-syndromic recessive deafness with enlarged vestibular aqueduct [5,6]. To date, more than 150 mutations in the *SLC26A4 *gene have been reported in patients with PDS or nonsyndromic deafness with enlarged vestibular aqueducts (http://www.healthcare.uiowa.edu/labs/pendredandbor/slcMutations.htm). According to previous reports, the H723R missense substitution accounts for up to 75% of *SLC26A4 *mutations in Japanese families with EVA [6,7]. There are many cases without goiter associated with the H723R mutation [3]. Madeo *et al*. found that thyroid gland volume is primarily *SLC26A4 *genotype-dependent in children but is age-dependent in adults [8]. These reports suggest that the variable degree of thyroid dysfunction and goiter associated with *SLC26A4 *mutations may be caused by factors unrelated to *SLC26A4 *genotype. It is noteworthy that reported homozygotes for the H723R mutation were mainly from Japan and Korea where daily iodine intake should be comparatively high [3,7,9]. We therefore hypothesized that the amount of iodine intake influences the thyroid phenotype associated with PDS, leading us to study the effect of dietary iodine deficiency on thyroid gland structure and function in *Slc26a4*-null mutant mice.

TT4 levels were lower in the ICD group than in the CCD group. This difference was observed regardless of genotype, and these results suggest the thyroid function of *Slc26a4*^-/- ^mice is approximately the same as of *Slc26a4*^+/- ^and *Slc26a4*^+/+ ^mice. While we were preparing the manuscript, we found a similar report by Calebiro *et al *[10]. In their report they also confirmed that dietary iodine restriction did not induce goiter in *Slc26a4*^-/- ^mice. However, Calebiro *et al*. reported that total TT4 levels did not differ significantly between mice fed a low-iodine diet in comparison to those fed a standard diet [10].

The reason why TT3 levels did not decrease might be because incompletely iodinated thyroglobulin (Tg) in the thyroid colloid is accompanied by an increase in monoiodotyrosine (MIT) on Tg molecules, resulting in preferential T3 synthesis [11]. Therefore, TT3 levels may have been maintained despite the decline in TT4 levels in iodine-deficient mice. Another explanation why TT3 was unchanged in mice fed an iodine-deficient diet is an increase of type 1 iodothyronine 5'-deiodinase (D1) activity in the thyroid gland. Pedraza *et al*. reported that thyroidal D1 activity was increased with an iodine-deficient diet [12].

Other factors may compensate for defective iodine transport in both patients with PDS and *Slc26a4*^-/- ^mice. Van den Hove *et al*. have reported that the ClCn5 (chloride channel 5) protein localizes at the apical membrane of thyrocytes. The thyroidal phenotype in ClCn5-deficient mice is similar to that in Pendred syndrome, suggesting that ClCn5 could participate in mediating apical iodine efflux or iodine/chloride exchange [13,14]. Suzuki *et al*. reported that thyroglobulin, by mediating differential expression of several thyroid-specific genes including *TSHR*, *NIS*, and *TPO*, *TG*, *PAX8*, *TTF1*, and *TTF2 *regulates the rate of iodide efflux into the follicular lumen and may thus play an important role in regulating thyroid function under constant levels of TSH [13,15].

In conclusion, the ICD did not induce goiter in *Slc26a4*-null mice whereas, in humans, *SLC26A4 *mutations sometimes lead to goiter and even hypothyroidism. Mice may be different from humans in their ability to transport iodide into the follicular lumen or mice may respond differently to altered iodine availability. It is also possible that our results result from the use of male experimental animals since goiter and hypothyroidism are more prevalent among human females than males. The genetic strain background may also influence the penetrance and expressivity of the thyroid phenotype associated with *Slc26a4 *mutations. These may be some of the factors involved in the development of goiter in PDS.

## Competing interests

The authors declare that they have no competing interests.

## Authors' contributions

TI designed and coordinated the study, performed the experiments and drafted the manuscript; TY and MT supervised all experimental procedures, participated in performing experiments, and helped to draft the manuscript; YM participated in coordination of the study and helped to draft the manuscript; YH participated in performing experiments. YK participated in coordination of the study. TN and AJG, the senior author, drafted the manuscript. All authors have read and approved the final manuscript.

## References

[B1] Kopp P Pesce L Solis-S J Pendred syndrome and iodide transport in the thyroid Trends Endocrinol Metab 2008 19 260 268 10.1016/j.tem.2008.07.001 18692402

[B2] Everett L Belyantseva I Noben-Trauth K Cantos R Chen A Thakkar S Hoogstraten-Miller S Kachar B Wu D Green E Targeted disruption of mouse Pds provides insight about the inner-ear defects encountered in Pendred syndrome Hum Mol Genet 2001 10 153 161 10.1093/hmg/10.2.153 11152663

[B3] Sato E Nakashima T Miura Y Furuhashi A Nakayama A Mori N Murakami H Naganawa S Tadokoro M Phenotypes associated with replacement of His by Arg in the Pendred syndrome gene Eur J Endocrinol 2001 145 697 703 10.1530/eje.0.1450697 11720893

[B4] Kanou Y Hishinuma A Tsunekawa K Seki K Mizuno Y Fujisawa H Imai T Miura Y Nagasaka T Yamada C Ieiri T Murakami M Murata Y Thyroglobulin gene mutations producing defective intracellular transport of thyroglobulin are associated with increased thyroidal type 2 iodothyronine deiodinase activity J Clin Endocrinol Metab 2007 92 1451 1457 10.1210/jc.2006-1242 17244789

[B5] Everett LA Glaser B Beck JC Idol JR Buchs A Heyman M Adawi F Hazani E Nassir E Baxevanis AD Sheffield VC Green ED Pendred syndrome is caused by mutations in a putative sulphate transporter gene (PDS) Nat Genet 1997 17 411 422 10.1038/ng1297-411 9398842

[B6] Usami S Abe S Weston M Shinkawa H Van Camp G Kimberling W Non-syndromic hearing loss associated with enlarged vestibular aqueduct is caused by PDS mutations Hum Genet 1999 104 188 192 10.1007/s004390050933 10190331

[B7] Kitamura K Takahashi K Noguchi Y Kuroishikawa Y Tamagawa Y Ishikawa K Ichimura K Hagiwara H Mutations of the Pendred syndrome gene (PDS) in patients with large vestibular aqueduct Acta Otolaryngol 2000 120 137 141 10.1080/000164800750000775 11603758

[B8] Manichaikul A Reynolds J Sarlis NJ Pryor SP Shawker T Griffith AJ Evaluation of the thyroid in patients with hearing loss and enlarged vestibular aqueducts Arch Otolaryngol Head Neck Surg 2009 135 670 676 10.1001/archoto.2009.66 19620588 PMC2941509

[B9] Park H-J Shaukat S Liu X-Z Hahn SH Naz S Ghosh M Kim H-N Moon S-K Abe S Tukamoto K Riazuddin S Kabra M Erdenetungalag R Radnaabazar J Khan S Pandya A Usami S-I Nance WE Wilcox ER Riazuddin S Griffith AJ Origins and frequencies of SLC26A4 (PDS) mutations in east and south Asians: global implications for the epidemiology of deafness J Med Genet 2003 40 242 248 10.1136/jmg.40.4.242 12676893 PMC1735432

[B10] Calebiro D Porazzi P Bonomi M Lisi S Grindati A De Nittis D Fugazzola L Marinò M Bottà G Persani L Absence of primary hypothyroidism and goiter in Slc26a4 (-/-) Mice Fed on a Low Iodine Diet J Endocrinol Invest 2010 20834201 10.3275/7262

[B11] Delange F The disorders induced by iodine deficiency Thyroid 1994 4 107 128 10.1089/thy.1994.4.107 8054857

[B12] Pedraza PE Obregon MJ Escobar-Morreale HF del Rey FE de Escobar GM Mechanisms of adaptation to iodine deficiency in rats: thyroid status is tissue specific. Its relevance for man Endocrinology 2006 147 2098 2108 10.1210/en.2005-1325 16455775

[B13] Bizhanova A Kopp P Genetics and phenomics of Pendred syndrome Mol Cell Endocrinol 2010 322 83 90 10.1016/j.mce.2010.03.006 20298745

[B14] van den Hove M Croizet-Berger K Jouret F Guggino S Guggino W Devuyst O Courtoy P The loss of the chloride channel, ClC-5, delays apical iodide efflux and induces a euthyroid goiter in the mouse thyroid gland Endocrinology 2006 147 1287 1296 16306076 10.1210/en.2005-1149

[B15] Suzuki K Kohn LD Differential regulation of apical and basal iodide transporters in the thyroid by thyroglobulin J Endocrinol 2006 189 247 255 10.1677/joe.1.06677 16648292

